# 121 The effectiveness and acceptability of physical activity interventions amongst older adults from socioeconomically deprived groups: A mixed methods systematic review

**DOI:** 10.1093/eurpub/ckae114.112

**Published:** 2024-09-26

**Authors:** Danielle Harris, Schenelle Dlima, Ashley Gluchowski, Alex Hall, Luke Munford

**Affiliations:** School of Health Sciences, University of Manchester, Manchester, United Kingdom; School of Health Sciences, University of Manchester, Manchester, United Kingdom; School of Health & Society, University of Salford, Salford, United Kingdom; School of Health Sciences, University of Manchester, Manchester, United Kingdom; School of Health Sciences, University of Manchester, Manchester, United Kingdom; NIHR Applied Research Collaboration-Greater Manchester (ARC-GM), Manchester, United Kingdom

## Abstract

**Purpose:**

Socioeconomically deprived older adults are less active compared to older adults who experience less socioeconomic deprivation. However, there is a paucity of evidence as to whether existing physical activity interventions are appropriate for older adults from lower socioeconomic groups. This review explores: (1) How effective are interventions aimed at increasing physical activity for older adults from socioeconomically deprived groups?; (2) What factors are associated with the acceptability of physical activity interventions amongst older adults from socioeconomically deprived groups?; (3) What are the implications for developing physical activity interventions for older adults from socioeconomically deprived groups?

**Methods:**

Nine databases were searched up to May 2023, to identify quantitative, qualitative, and mixed methods primary studies measuring the effectiveness and/or acceptability of physical activity interventions amongst older adults (aged 65 years and over) from socioeconomically deprived groups. Study authors’ definitions of socioeconomic deprivation were accepted; studies which described participants as socioeconomically deprived/disadvantaged or lower socioeconomic status were included. Included studies were assessed for quality using the Mixed Methods Appraisal Tool. This review follows a convergent segregated data synthesis approach.

**Results:**

Thirty studies (18 quantitative, 6 qualitative and 6 mixed methods) with 6,414 total participants were included. Mixed effects were found for effectiveness of interventions on physical activity levels amongst older adults from socioeconomically deprived groups. Effective interventions were informed by behaviour change frameworks, most commonly Social Cognitive Theory, utilised behaviour change techniques including goal-setting and self-monitoring and delivered in community settings, mainly via supervised group sessions.

**Conclusions:**

The findings from this review will increase understanding around what makes physical activity interventions effective and acceptable to older adults from lower socioeconomic groups to inform future development of targeted, appropriate interventions.

**Support/Funding Source:**

This presentation presents independent research funded under the Dunhill Medical Trust (PDM2202\9), National Institute for Health and Care Research Applied Research Collaboration-Greater Manchester (NIHR20017405156), National Institute for Health and Care Research Policy Research Unit in Older People and Frailty (PR-PRU-1217-21502) / Healthy Ageing (NIHR206119) and the University of Manchester. The views expressed are those of the author(s) and not necessarily those of the NIHR or Department of Health and Social Care.

